# 

**Published:** 1906-05-26

**Authors:** 


### Medical Appointments and Vacancies.

### VACANCIES.

Cancer Hospital, Fulham Road, London, S.W.—Surgical Registrar.

Central London Ophthalmic Hospital, Gray's Inn Road, W.C.—House Surgeon. Also Pathologist

Charing Cross Hospital.—Assistant Physician.

Chelsea Hospital for Women, Fulham Road, London, S.W. Surgeon.

Evelina Hospital for Sick Children, Southwark.—House Physician and House Surgeon. Also Assistant House Surgeon.

Exeter, Royal Devon and Exeter Hospital.—Assistant House Surgeon.

Huddersfield Infirmary.—Senior House Surgeon.

Hull, Royal Infirmary.—House Physician. Also Casualty House Surgeon.

North-Eastern Hospital for Children, Hackney Road, Bethnal Green, E.—House Surgeon.

North-West London Hospital, Kentish Town Road.—Resident Medical Officer and Assistant Resident Medical Officer.

Parish of Paddington.—District Medical Officer.

Perth District Asylum, Murthly.—Assistant Physician.

Royal College of Surgeons of England.—Hunterian Professors, Erasmus Wilson Lecturer, and Arris and Gale Lecturer.

University College, London.—Assistant Dental Surgeon.

Western General Dispensary, Marylebone Road, N.W.—Honorary Surgeon.

### BOOKS RECEIVED.

Baillière, Tindall and Cox.

"Diseases of the Eye," by C. H. May, M.D., and C. Worth, F.R.C.S.

"The Nature and Treatment of Cancer," 3rd edition, by J. A. Shaw-Mackenzie, M.D.

"The Cause and Prevention of Dental Caries," by J. Sim Wallace, M.D.

Cassell and Co., Ltd.

"Advice to Women," by Florence Stackpoole.

F. A. Davis Co., Philadelphia.

"Auto-Intoxication in Disease," by Professors C. H. Bouchard and T. Oliver.

"THE HOSPITAL" says:—

"Every Hospital Matron, Steward, and Housekeeper should possess **that excellent book.**"

### HOSPITAL EXPENDITURE

2/6 post free. **The Commissariat** 2/6 post free.

A close and careful study of this work will enable Hospital Managers to add largely to their reputation for economy and efficiency.

### THE COTTAGE HOSPITAL CASE-BOOK, OR REGISTER OF PATIENTS

Prepared and ruled in accordance with what has been found in practice to be the most approved system, and suitable for large or small Cottage Hospitals. Bound in half basil, green cloth.

IN TWO SIZES:—

10/- net, 10/3 post free. No. I., containing space for about 150 in-patient cases, with rulings for 8 weeks' payments to each case. 10/- net, 10/3 post free.

12/6 net, 12/10 post free. No. II., containing space for about 300 in-patient cases, with rulings for 8 weeks' payments to each case. 12/6 net, 12/10 post free.

### THE FURNISHING AND APPLIANCES OF A COTTAGE HOSPITAL

**An Actual Inventory.**

6d. net, 7d. post free. By Hon. SYDNEY HOLLAND. 6d. net, 7d. post free.

### HOSPITAL AND PRIVATE NURSING

**The Matron's Course.**

1/- post free. By Miss S. E. ORME. 1/- post free.

### SYLLABUS OF LECTURES TO NURSES

1/- post free. By ANDREW DAVIDSON, M.D. 1/- post free.

### HOSPITAL SISTERS AND THEIR DUTIES

2/6 post free. By EVA C. E. LUCKES, Matron, London Hospital. 2/6 post free.

**The Scientific Press, Ltd.,**

*Catalogue of Nursing Manuals sent post free on application.*

**Publishers of Nursing Text-Books, Charts, and Case-Books of all Descriptions.**

**28 & 29 SOUTHAMPTON STREET, STRAND, LONDON, W.C.**

### Publications.

'JUST PUBLISHED. Third Edition, Revised and Enlarged, with Additional Matter. Price 2s. 6d. net.

**THE NATURE AND TREATMENT OF CANCER.**

(Some Methods of Hypodermic Medication in the Treatment of Inoperable Cancer).

By JOHN A. SHAW-MACKENZIE, M.D. (Lond.).

Medical Press and Circular, when reviewing the second edition, said :—" So greatly have we been impressed by the perusal of this work that we can only recommend every practitioner to study it."

London : BAILLIÈRE, TINDALL, & COX, 8 Henrietta Street, Covent Garden.

**DISEASES OF THE ANUS & RECTUM**

By D. H. GOODSALL, F.R.C.S., and W. ERNEST MILES, F.R.C.S.,

Senior Surgeon Metropolitan Hospital; late Senior Surgeon, St. Mark's Hospital. Surgeon Cancer Hospital; Surgeon Gordon Hospital.

PART I.—Containing Chapters on Anatomy, General Diagnosis, Abscess, Ano-Rectal Fistula, Recto-Urethral, Recto-Vaginal and Recto-Vesical Fistula, Sinus over the Sacrum and Coccyx, Anal Fissure and Hæmorrhoids (or Piles). With 91 Illustrations, 8vo. 7s. 6d. net.

PART II.—Containing Chapters on Prolapse, Invagination Ulceration, Stricture, Malignant Growths, Benign Tumours, Foreign Bodies, Pruritis Ani, and Syphilis. With 44 Illustrations, 8vo. 6s. net.

LONGMANS, GREEN & CO., London, New York, Bombay.

**WORKS BY DAVID WALSH, M.D.**

RÖNTGEN RAYS IN MEDICAL WORK. *Third Edition.* 12s. 6d.

THE HAIR AND ITS DISEASES. 2s. 6d. net. A Standard Monograph. Baillière, Tindall & Cox, London.

GOLDEN RULES OF SKIN PRACTICE. 1s. net. *Second Edition.* Wright & Co., Bristol.

AGE AND OLD AGE: Handbook. 2s. 6d. R. A. Everett & Co. London.

JUST PUBLISHED. Price **2s. 6d.** net.

**CARCINOMA OF THE RECTUM.**

By F. SWINFORD EDWARDS, F.R.C.S.,
Surgeon to the West London Hospital; Senior Surgeon to St. Peter's Hospital for Stone, and to St. Mark's Hospital for Fistula, &c.

London : Baillière, Tindall, & Cox, 8 Henrietta Street, Covent Garden.

**"THE UNIFORM SYSTEM OF ACCOUNTS."**

By SIR HENRY BURDETT, K.C.B.

THE INDEX OF CLASSIFICATION

Whereby every item of expenditure may be dealt with under identical heads by every group of Institution is now published separately, price 1s.; 1s. 1d. post free.

THE SCIENTIFIC PRESS, LTD.,
28 and 29 Southampton Street, Strand, London, W.C.

**"An invaluable handbook for all engaged in philanthropic work."—St. James's Gazette.**

Over 1,000 pp. Price **6/-** net; **6/5** post free.

### Burdett's Hospitals & Charities, 1906.

BEING THE YEAR-BOOK OF PHILANTHROPY AND THE HOSPITAL ANNUAL.

Of all Booksellers, or of

**THE SCIENTIFIC PRESS, LTD., 28 & 29 Southampton Street, Strand, London, W.C.**

